# An Unusual Case of Thalamic Stroke in a Young Adult With Patent Foramen Ovale and Finasteride Use

**DOI:** 10.7759/cureus.60300

**Published:** 2024-05-14

**Authors:** Sai Rakshith Gaddameedi, Mahrukh A Khan, FNU Arty, Vandana Bandari, Anoohya Vangala, Pratik Panchal, Shazia M Shah

**Affiliations:** 1 Internal Medicine, Monmouth Medical Center, Long Branch, USA; 2 Internal Medicine, Rutgers Health/Monmouth Medical Center, Long Branch, USA; 3 Internal Medicine, Bayhealth Medical Center, Dover, USA; 4 Cardiology, Monmouth Medical Center, Long Branch, USA

**Keywords:** stroke, finasteride, thalamic stroke, patent foramen ovale, pfo

## Abstract

Symptomatic cerebral infarcts with cryptogenic ischemic stroke pose diagnostic challenges due to unknown etiology. Notably, up to half of young individuals with cryptogenic stroke exhibit patent foramen ovale (PFO), while finasteride, which is used for male pattern baldness, elevates testosterone levels, potentially increasing the risk of thrombosis. Here, we present a case of thalamic infarction in a 21-year-old male devoid of cerebrovascular risk factors but with PFO and finasteride use. The patient presented with short-term memory issues, otherwise lacking medical history or substance use. Examination revealed neurological deficits, with imaging indicating a left thalamic infarct. Subsequent investigations identified PFO, prompting referral for closure, yielding symptomatic improvement. Furthermore, discontinuation of finasteride was advised due to its thrombotic association. Finasteride's inhibition of 5-alpha reductase 2 increases testosterone conversion to estrogen, potentially promoting thrombosis. Finasteride use can cause thrombotic events, emphasizing its risk. In conclusion, young embolic stroke patients warrant PFO evaluation alongside hypercoagulable workup, with closure benefiting those under the age of 55. Additionally, discontinuing finasteride may mitigate thrombosis risk.

## Introduction

Symptomatic cerebral infarcts with a cryptogenic ischemic stroke usually have no known plausible etiology identified even after a thorough diagnostic evaluation. One-quarter of patients with ischemic stroke have no probable cause found after standard workup [1]. Up to half of young individuals with cryptogenic stroke had patent foramen ovale (PFO) [2]. A PFO is the most frequent reason for a right-to-left shunt. Although it usually closes within three months of birth, this interatrial defect may remain open throughout life, and if it does, venous thromboemboli may be able to evade filtration in the pulmonary vasculature and enter the systemic arterial circulation, increasing the risk of a cryptogenic stroke [3].

An FDA-approved pharmaceutical agent, finasteride, treats male pattern hair loss and benign prostatic hyperplasia in males. Finasteride inhibits testosterone's conversion to dihydrotestosterone by acting as a competitive inhibitor of types II and III of the 5-alpha-reductase enzyme (DHT) [4]. The Japan Pharmaceutical and Medical Devices Agency (PMDA) has documented 14 occurrences of thrombosis in finasteride-using individuals; these cases included six cases of myocardial infarctions, four cases of other thrombotic disorders, and two cases of strokes. The hypothesis is that increases in estrone and estradiol levels due to finasteride use may be implicated in the development of thrombosis [5]. Here, we report a case of a young adult male with an unusual presentation of a thalamic infarct without cerebrovascular disease risk factors but with two potentially contributory but controversial etiologies: PFO and finasteride use.

## Case presentation

A 21-year-old Caucasian male with no known medical history presented to the emergency department (ED) for the evaluation of memory issues. One day prior to presentation, he noticed that he was not able to remember certain numbers and had issues with his memory. The following morning, he left for work; however, he could not remember the code to enter this facility, at which point he realized that something serious is going on, after which he presented to the ED. The patient denied any fever or chills in the past couple of weeks. However, he reported a distant history of flulike illness a month back. The patient reported exercising regularly and denied smoking alcohol drug use. He has been using finasteride since a year for male pattern baldness.

In the ED, his blood pressure was 103/57, heart rate was 77 beats/min, respiratory rate was 18 breaths/min, and saturation was 97% on room air, and he was afebrile. During the examination, he was slow to react and his speech was pressured; however, he was able to answer all the questions. He was alert, awake, and oriented; neurologic examination revealed short-term memory gaps but no long-term memory issues or signs of cerebellar dysfunction. He had no weakness, sensory or motor deficits, abnormal gait, abnormal deep tendon reflexes, or abnormal coordination, and his cranial nerves were grossly intact. His complete blood count, comprehensive metabolic panel, and urine toxicology screening were within normal limits.

**Figure 1:**
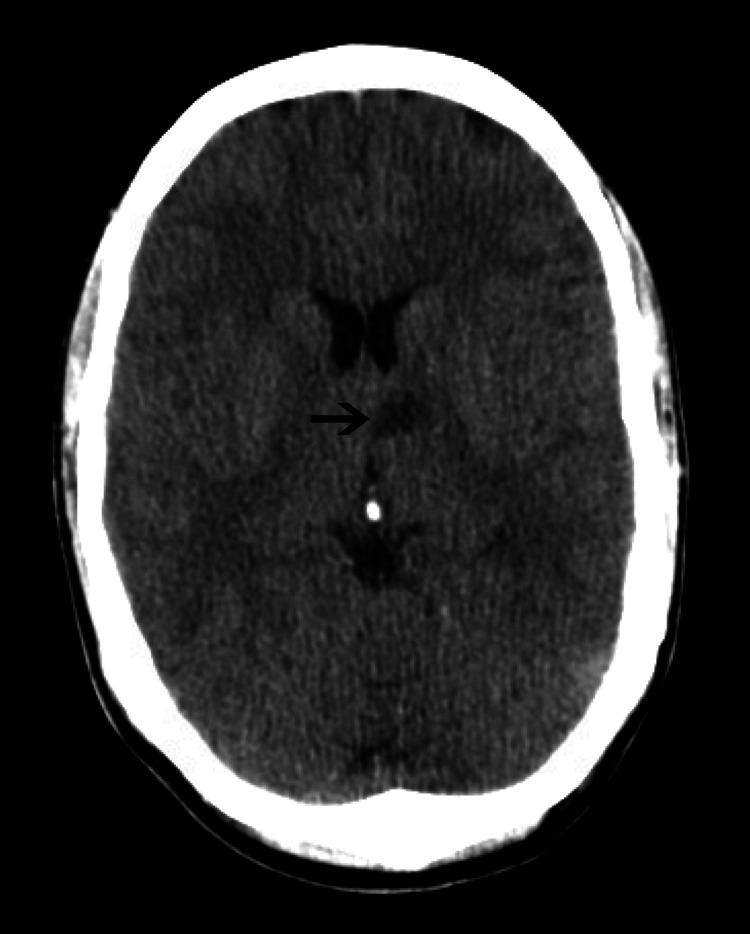
CT of the head showing subacute infarct on the left thalamus

**Figure 2:**
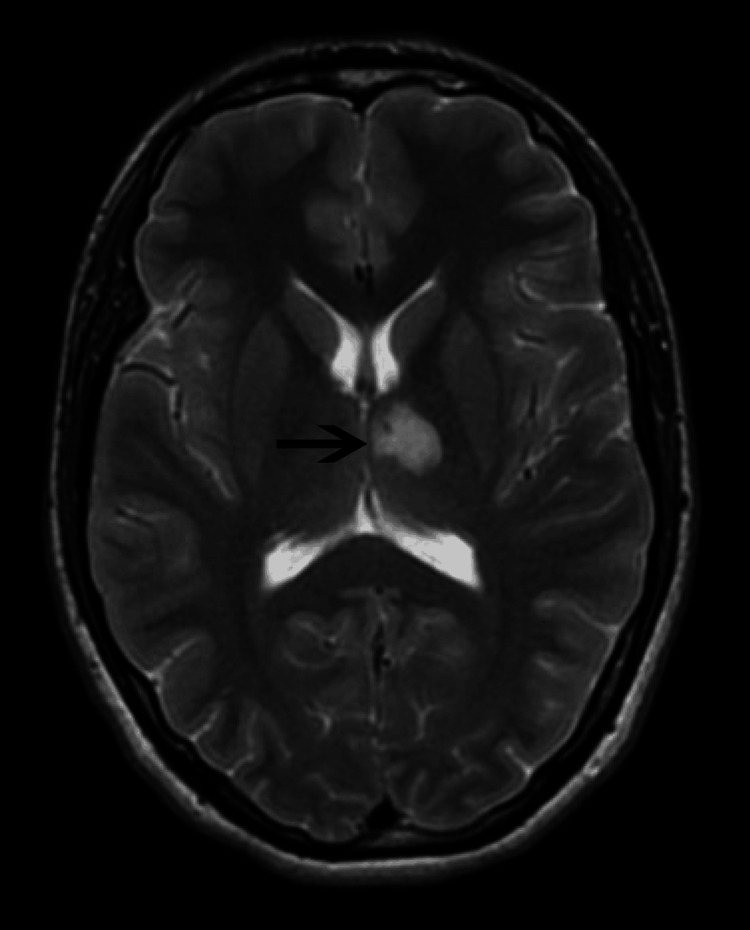
MRI of the brain showing an acute left thalamic infarct

Computed tomography scan of the head (Figure 1) showed a hypoattenuating left thalamic lesion concerning for a subacute infarct versus a demyelinating lesion. Immediately, the stroke team was alerted and neurology was consulted, and the patient was started on aspirin, clopidogrel, and statin. Cardiology was consulted for the evaluation of embolic source of stroke, and hypercoagulable workup was ordered. Magnetic resonance imaging (MRI) (Figure 2) revealed restricted diffusion of the left thalamus, consistent with an acute infarct. Magnetic resonance angiography of the head and neck was normal. Venous Doppler ultrasound and computed tomography of pulmonary angiography were normal. His coagulation workup was negative for factor V Leiden, protein C, protein S, lupus anticoagulant screen, and anti-thrombin. Lyme serology and HIV (human immunodeficiency virus) were also negative. Cardiology performed a transthoracic echocardiography, which was normal, but due to a high suspicion of an embolic stroke, a transesophageal echocardiography was performed, which revealed PFO, and the injection of contrast documented an interatrial shunt. As the patient was young and active, a decision was made to refer him for PFO closure.

The patient later had a successful PFO closure and was asymptomatic on outpatient follow-up with cardiology. He was also advised to stop using finasteride due to its probable association with stroke.

## Discussion

The percentage of young adults experiencing their first stroke varies by country and ranges from 5% to 20% of all strokes [6]. In community-based and population-based research, it has been found that men had a higher incidence of stroke in young people than women older than 35 years [7]. The three most prevalent vascular risk factors in young individuals were hypertension (44 %), smoking (60 %), and dyslipidemia (60 %) (39 %). Twenty to thirty percent of youth stroke cases are due to strokes with other known etiologies. Up to one-third of ischemic strokes in young individuals are attributed to cardioembolic stroke [8].

Cardioembolic stroke is often more difficult to diagnose. The causes of cardiogenic embolism are divided into major and minor sources by Hart [9]. Major risk factors were atrial fibrillation, prosthetic heart valves, myocardial infarction (MI), intracardiac thrombus, mitral stenosis, atrial myxoma, dilated cardiomyopathy, left ventricular aneurysm, and infective endocarditis. Minor risk factors were PFO, recent atrial septal aneurysm, mitral valve prolapse, spontaneous echo contrast, mitral annular calcification, calcific aortic stenosis, and ventricular akinesia [9].

The role of the PFO in cardioembolism is one of the topics that is frequently up for discussion. PFO is around 20% more common in young stroke patients than it is in the general population (up to 50%) [10]. The union of the septum primum and septum secundum closes the foramen ovale during the first year of life. The PFO, which resembles an interatrial slit, results when this process fails [11,12]. Around 15-40% of all ischemic strokes are cryptogenic strokes, and 40-56% of patients under the age of 55 who have cryptogenic stroke or a transient ischemic attack have PFO [13]. Young age, PFO size, right-to-left shunt degree, PFO morphology, presence of atrial septal aneurysm, intrinsic coagulation-anticoagulation systems imbalance, and coexistence of other atrial abnormalities, such as right atrial septal pouch, Eustachian valve, and Chiari's network, are the main risk factors associated with PFO-attributable strokes [14].

The RoPE score, a rating that considers factors such as youth, the location of the cortical infarct, and the absence of conventional stroke risk factors, is connected to the likelihood that a PFO is pathogenic and the chance of having another stroke after the initial one [15]. The presence of PFO should be taken into consideration as an etiological suspect in young individuals with cryptogenic stroke. It is important to use caution when determining the significance of other PFO traits [16].

In this case, the only medication that the patient was taking was finasteride. Androgenetic alopecia is recognized by a predictable pattern of androgen-related progressive hair thinning. Regardless of age or stage of baldness, it causes diminished self-esteem, reduces confidence, and causes unhappiness in affected males. The enzyme 5-reductase type 2, which transforms testosterone into its more potent version, DHT, is inhibited by finasteride. High-level data support finasteride's orally administered effects on men's ability to develop hair [17]. The Japan Pharmaceutical and Medical Devices Agency (PMDA) has documented 14 instances of thrombotic events among individuals using finasteride in Japan. These include four cases of stroke, six cases of myocardial infarction, and four cases of other thrombotic conditions. Finasteride raises the levels of peripherally aromatized testosterone in the blood, which is converted to estrogens. Finasteride-induced elevations in estrone and estradiol levels have been implicated in thrombus development. Young adults who suffer strokes may experience an increase because of this [5].

## Conclusions

In conclusion, the case presented underscores the complexities inherent in diagnosing and managing strokes, particularly in young individuals with cryptogenic etiologies. The presence of PFO, although controversial in its role in stroke pathogenesis, warrants consideration in cases of cryptogenic stroke, especially in young patients. The decision to refer for PFO closure in this case reflects the evolving understanding of its significance and the potential benefits of intervention in reducing recurrent stroke risk. Furthermore, the association between finasteride use and thrombosis, while not fully elucidated, merits attention. We suspect a potential correlation between the use of finasteride and stroke, and we wish to highlight this association as likely being a strong risk factor for thrombosis especially in cases like this one.

## References

[1] Saver JL (2016). Cryptogenic stroke. N Engl J Med.

[2] Mojadidi MK, Zaman MO, Elgendy IY (2018). Cryptogenic stroke and patent foramen ovale. J Am Coll Cardiol.

[3] Gonnah AR, Bharadwaj MS, Nassar H, Abdelaziz HK, Roberts DH (2022). Patent foramen ovale: diagnostic evaluation and the role of device closure. Clin Med (Lond).

[4] Zito PM, Bistas KG, Syed K (2024). Finasteride. StatPearls [Internet].

[5] Tsuji Y, Nakayama T, Bono K, Kitamura M, Imafuku I (2014). [Two cases of stroke associated with the use of finasteride, an approved drug for male-pattern hair loss in Japan] [Article in Japanese]. Rinsho Shinkeigaku.

[6] Nedeltchev K, der Maur TA, Georgiadis D (2005). Ischaemic stroke in young adults: predictors of outcome and recurrence. J Neurol Neurosurg Psychiatry.

[7] Lauria G, Gentile M, Fassetta G (1995). Incidence and prognosis of stroke in the Belluno province, Italy. First-year results of a community-based study. Stroke.

[8] Smajlović D (2015). Strokes in young adults: epidemiology and prevention. Vasc Health Risk Manag.

[9] Hart RG (1992). Cardiogenic embolism to the brain. Lancet.

[10] Alsheikh-Ali AA, Thaler DE, Kent DM (2009). Patent foramen ovale in cryptogenic stroke: incidental or pathogenic?. Stroke.

[11] Aggeli C, Verveniotis A, Andrikopoulou E, Vavuranakis E, Toutouzas K, Tousoulis D (2018). Echocardiographic features of PFOs and paradoxical embolism: a complicated puzzle. Int J Cardiovasc Imaging.

[12] Hagen PT, Scholz DG, Edwards WD Incidence and size of patent foramen ovale during the first 10 decades of life: an autopsy study of 965 normal hearts. Mayo Clinic Proc.

[13] Melkumova E, Thaler DE (2017). Cryptogenic stroke and patent foramen ovale risk assessment. Interv Cardiol Clin.

[14] Ioannidis SG, Mitsias PD (2020). Patent foramen ovale in cryptogenic ischemic stroke: direct cause, risk factor, or incidental finding?. Front Neurol.

[15] Kent DM, Ruthazer R, Weimar C (2013). An index to identify stroke-related vs incidental patent foramen ovale in cryptogenic stroke. Neurology.

[16] Overell JR, Bone I, Lees KR (2000). Interatrial septal abnormalities and stroke: a meta-analysis of case-control studies. Neurology.

[17] Manabe M, Tsuboi R, Itami S (2018). Guidelines for the diagnosis and treatment of male-pattern and female-pattern hair loss, 2017 version. J Dermatol.

